# A Comparative Study of Machine Learning and Deep Learning Models for Long-Term Snow Depth Inversion

**DOI:** 10.3390/s26041220

**Published:** 2026-02-13

**Authors:** Tingyu Lu, Rong Fan, Lijuan Zhang, Qiang Wang, Yufeng Zhao, Lei Wang, Yutao Huang

**Affiliations:** 1 Heilongjiang Institute of Technology, College of Surveying and Mapping Engineering, Harbin 150050, China; lutingyu@hljit.edu.cn (T.L.); wangqiang310108@aliyun.com (Q.W.); wanglei@hljit.edu.cn (L.W.); 2 College of Geographical Sciences, Harbin Normal University, Harbin 150025, China; fanrong1055@163.com (R.F.); janekabesilsen@163.com (Y.Z.); huangyutao0128@163.com (Y.H.)

**Keywords:** snow depth inversion, machine learning, deep learning, long time series, meteorological factors, snow physical parameters

## Abstract

Snow depth is a critical parameter for characterizing snow dynamics and water resources, and its accurate inversion is essential for hydrological processes, climate studies, and disaster prevention in cold regions. Based on long-term daily ground meteorological observation data from the hydrological years 1961 to 2015 at two meteorological stations in Mohe and Mishan, Heilongjiang Province, China, this study integrates physical parameters of snow density and snow albedo from the ERA5-Land reanalysis data to systematically compare the performance of three machine learning and three deep learning models in retrieving daily snow depth. Four feature combination schemes were designed to evaluate the contributions of meteorological factors, lagged snow depth terms, and snow physical parameters. The results indicate that, for both machine learning and deep learning models, the first-order lagged value of snow depth is the most important variable determining prediction accuracy. In terms of model performance, machine learning methods excelled, with XGBoost performing particularly outstandingly, achieving optimal prediction accuracy and stability under the best feature combination (coefficient of determination, R^2^, reaching 0.989; root mean square error, RMSE, of 1.19 cm). Among deep learning methods, 1D CNN demonstrated strong local feature extraction capabilities, achieving prediction accuracy comparable to the best-performing machine learning model (R^2^ of 0.9878, RMSE of 1.26 cm). Notably, models specifically designed for time-series data, such as LSTM (R^2^ of 0.9848, RMSE of 1.41 cm), and the more complex 1D CNN-LSTM hybrid model (R^2^ of 0.9803, RMSE of 1.60 cm) did not show significant advantages in this study. This indicates that model complexity and predictive performance are not simply positively correlated. Through comprehensive analysis of data from both stations, this study demonstrates that a prediction framework centered on historical snow depth as the core driving factor, combined with key meteorological elements, is highly robust. Although the inclusion of ERA5-Land snow physical parameters did not significantly improve model accuracy, it provides important insights for the future development of hybrid models that integrate physical mechanisms with data-driven approaches. The findings offer an effective solution for reconstructing long-term snow depth time series and hold significant application value for simulating cryospheric hydrological processes and studying climate change.

## 1. Introduction

Snow cover plays a crucial role in the global climate system, water cycle, ecological environment, and socioeconomic activities. Its dynamic changes profoundly impact the balance of the natural environment and the well-being of human society. At the 1972 United Nations Conference on Environment and Development, the World Meteorological Organization (WMO) first introduced the concept of the “cryosphere”. Today, the cryosphere has evolved into the fifth major sphere alongside the biosphere, hydrosphere, atmosphere, and lithosphere. It has become an essential component of the global climate system and plays a pivotal role in climate change research [1,2]. Snow cover refers to the layer of snow accumulated on the surfaces of land and sea ice. It exhibits high sensitivity to climate change and exerts significant feedback effects, making it a vital part of the climate system. Changes in snow cover not only profoundly influence energy balance and hydrological processes but also serve as a sensitive indicator of climate change [3]. On a global scale, snow cover, with its high albedo and extensive cooling effects, plays a significant regulatory role in regional and even global climate by modulating land-atmosphere energy exchange. In terms of positive impacts, snowmelt runoff provides a critical water source for ecosystems, particularly important for water resource replenishment in arid and semi-arid regions. Meanwhile, snow water equivalent, as a key parameter in meteorological, climatic, and hydrological models, holds significant value for improving the accuracy of water resource assessments and weather forecasting capabilities. However, changes in snow cover also come with non-negligible potential risks. Heavy snowfall, persistent low temperatures, and prolonged snow cover can trigger snow disasters, directly affecting livestock production, while rapid snowmelt may lead to soil erosion and increased flood risks, posing threats to both the ecological environment and socioeconomic stability [4,5]. Through its unique physical properties and phase-change processes, snow cover plays a dual regulatory role in the global climate system. Its trends and spatial patterns hold important implications for climate prediction, water resource management, and disaster prevention and control [6].

Currently, the acquisition of snow cover parameters primarily relies on two categories of observation and simulation techniques: direct and indirect. Direct observations include ground-based measurements from stations (instrumental monitoring and manual sampling) and remote sensing observations. Indirect methods encompass process-based model simulations grounded in physical processes and reanalysis products that integrate multi-source data [7,8,9]. Spaceborne passive microwave remote sensing, with its unique advantages of cloud penetration and all-weather operation, has become a cornerstone for monitoring snow cover parameters at global and hemispheric scales. Since the 1970s, sensors such as the SMMR aboard the Nimbus-7 satellite, SSM/I and SSMIS on the DMSP series, AMSR-E on the Aqua satellite, AMSR-2 on the GCOM-W1 satellite, and MWRI on the Fengyun series have formed a complete passive microwave observation sequence, providing a long-term data foundation for snow cover research [10,11,12,13,14]. Regarding inversion algorithms, the Chang algorithm, based on the principle of brightness temperature difference, established a linear relationship between the horizontal polarization brightness temperature difference at 18 GHz and 36 GHz and snow depth, marking a milestone in passive microwave snow depth inversion [7]. Subsequent research has continuously improved algorithmic accuracy by incorporating polarization differences, adjusting regional coefficients, and accounting for forest coverage and topographic factors [15,16,17,18,19]. With the deepening understanding of snow’s physical properties, indices like the temperature gradient index and dense media radiative transfer models have further enhanced algorithm sensitivity to snow characteristics such as grain size, density, and stratigraphy [20,21]. Although radiative transfer models offer superior mechanistic representation, the difficulty in obtaining their required parameters limits their operational application. Current research is focused on constructing more accurate snow depth inversion models by integrating static geographical factors with multivariate meteorological data, thereby providing crucial data support for climate change studies and water resource management [22,23].

Snow research based on optical satellites has become a critical method for monitoring global snow cover dynamics, leading to the development of multiple generations of globally influential snow cover products. By utilizing data from satellites such as Landsat [24], SPOT [25], AVHRR [26], Sentinel-2 [27], and MODIS [28,29], researchers can accurately track snow cover extent, duration, and ablation processes. Among these, the MODIS sensors aboard the Terra and Aqua satellites are the most extensively studied and widely applied in this field. Their unique combination of high temporal resolution, moderate spatial resolution, and rich spectral bands provides an ideal data source for precisely extracting snow cover extent, identifying snow properties, and monitoring spatiotemporal dynamics. Combined with their released series of standardized datasets, including daily global snow cover products, snow albedo products, and snow grain size products, MODIS data constitutes one of the most comprehensive and essential data systems for current global and regional-scale snow cover monitoring and research.

Synthetic Aperture Radar (SAR) systems overcome the limitations imposed by illumination and weather conditions on optical sensors. Leveraging their active microwave detection capabilities, they demonstrate unique advantages in the quantitative inversion of snow cover parameters. Their penetration ability and the physical response mechanism of signals to the dielectric properties and layered structure of snow provide a theoretical foundation for snow depth detection distinct from that of optical remote sensing [30]. Sentinel-1 C-band data, with its high spatiotemporal resolution and free, open-access policy, is widely used for large-scale, high-frequency snow cover monitoring, showing particular strength in capturing wet snow dynamics [31]. In contrast, L-band SAR systems like PALSAR and PALSAR-2, with their stronger penetration capability, exhibit higher sensitivity to dry snow depth. Snow depth can be retrieved using techniques such as interferometric coherence or backscatter models [32].

Machine learning and deep learning algorithms, serving as core drivers in transforming the paradigm of remote sensing data analysis, have demonstrated significant potential in fields such as target recognition, land cover classification, and spatiotemporal prediction. In the estimation and inversion of snow cover parameters, these data-driven methods effectively circumvent the challenges faced by traditional physical models, which require explicit description and simplification of complex snow processes. Instead, they directly learn the intricate relationships between remote sensing observations and ground-based measurements of snow parameters. Compared to empirical linear regression algorithms that rely on brightness temperature differences, machine learning models like Support Vector Machines and Random Forests demonstrate superior capability in handling the “saturation effect” of snow and maintain higher robustness across diverse underlying surface conditions [33]. Furthermore, deep learning approaches, particularly recurrent neural networks such as LSTMs, introduce the ability to capture the temporal evolution characteristics of snow. By learning the dynamic processes of snow accumulation and ablation from long time-series data, they offer the potential for accurate snow depth prediction [34].

Snow depth, as a key parameter for characterizing snow dynamics and assessing water resources, holds significant importance for hydrological processes and climate research in cold regions. This study addresses the prediction of snow depth during the hydrological years from 1961 to 2015. By integrating ground-based meteorological observation data (including ground temperature, relative humidity, air temperature, wind speed, and sunshine duration) with key snow physical parameters—specifically snow density and snow albedo—from the ERA5-Land reanalysis dataset, it systematically applies various time-series-based machine learning and deep learning methods to construct snow depth prediction models that simultaneously account for meteorological drivers and snowpack physical states. The introduction of snow density and snow albedo, which have clear physical meanings, aims to enhance the model’s physical interpretability and improve the accuracy and stability of long-term snow depth predictions. The research focuses on investigating the synergistic influence mechanisms of meteorological factors and snow physical parameters, evaluating the performance of different time-series models in snow depth prediction, and elucidating the contribution mechanisms of snow density and snow albedo to simulation accuracy. Consequently, this study aims to provide a high-precision predictive framework for long-term snow depth reconstruction, one that integrates multi-source data with advanced time-series modeling approaches.

## 2. Study Area and Data

### 2.1. Study Area

**Figure 1:**
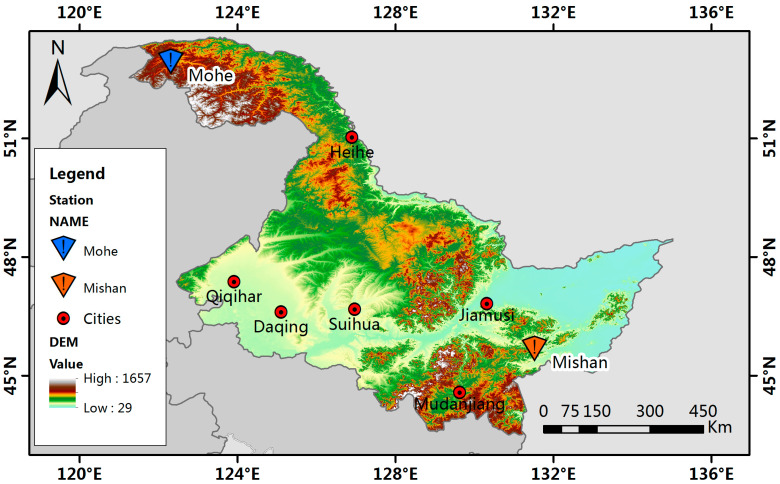
Location of the ground station.

Heilongjiang Province is located in northeastern China and is the northernmost and highest-latitude province in the country. Two typical monitoring stations with significant environmental differences were selected, providing a unique window for studying snow depth variations (see Figure 1). The Mohe station (52.58° N, 122.31° E) is situated within the city of Mohe, located in the Greater Khingan Range forest area at an elevation of 438.5 m. The terrain here is relatively rugged, and the land use is predominantly forested, representing the typical characteristics of a cold-temperate forest snow region. Mohe is known as the coldest county in China. Influenced by alternating continental and marine monsoons, the local microclimate is highly variable, with significant regional climatic differences. During winter, under the control of polar continental air masses, the climate is cold, dry, and prolonged. The Mishan monitoring station (45.33° N, 131.52° E) is located in the agricultural region of the Sanjiang Plain, at an elevation of 151.7 m. It experiences a mid-temperate continental monsoon climate, with agricultural land use and gentle terrain, representing the snow environment of a mid-latitude agricultural area. The relatively flat topography results in more uniform snow distribution [35].

### 2.2. Station Data

**Figure 2:**
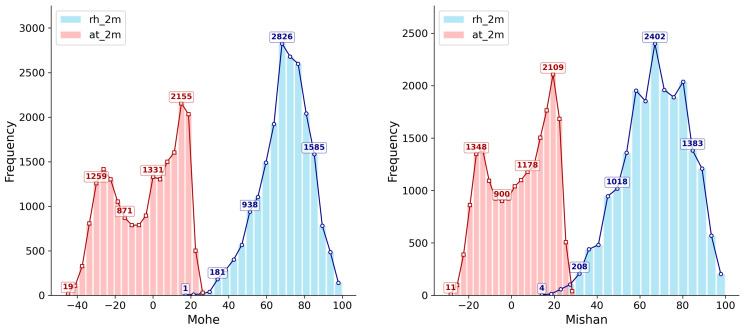
The frequency distribution characteristics of temperature and relative humidity at Mohe and Mishan weather stations.

The meteorological observation data used in this study cover the period from 1 August 1961, to 31 July 2016, forming a long-term data series comprising 20,089 days. Table 1 summarizes the statistics for temperature, relative humidity, and snow depth. The temperature in Mohe ranges from −46.7 °C to 28.1 °C, with an average of −4.09 °C, while in Mishan, the temperature ranges from −29.8 °C to 29.9 °C, with an average of 3.89 °C. Regarding relative humidity, the average in Mohe is 69.45%, and nearly half (49.2%) of the days maintain relative humidity in the high range of 70–90%. In Mishan, the frequency of days within this humidity range is 40.2%, indicating that Mohe is generally more humid. Additionally, the maximum recorded snow depth in Mohe is 53 cm, while in Mishan it is 41 cm. Figure 2 visually illustrates the distribution characteristics of relative humidity and temperature at the two sites using histograms, highlighting a clear climatic contrast between Mohe and Mishan, with Mohe exhibiting a colder and more humid climate pattern compared to Mishan.

**Figure 3:**
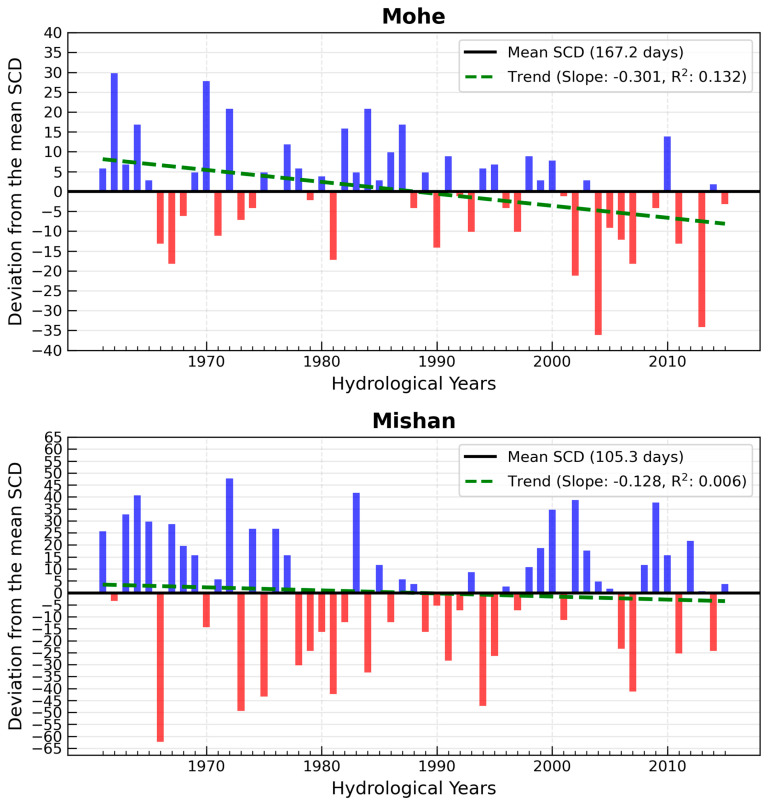
Analysis of trends in mean snow cover duration variation by hydrological year in Mohe and Mishan. Blue and red bars represent positive (increase) and negative (decrease) deviations from the mean snow cover days, respectively.

Figure 3 compares the snow cover duration (SCD) between the Mohe and Mishan meteorological stations from 1961 to 2015, revealing distinctly different snow cover characteristics at the two sites. Located at a higher latitude, Mohe receives less solar energy due to lower solar radiation angles, resulting in long and cold winters that support an extended and stable snow cover period. Its average annual SCD is 167 days, compared to 105 days in Mishan, indicating that Mohe’s SCD is approximately two months longer on average—a clear reflection of the significant latitudinal difference. Mohe is less influenced by oceanic moderation and is predominantly controlled by the Siberian cold high-pressure system in winter, leading to relatively small interannual climate variability. Consequently, its snow cover days are exceptionally stable, with a coefficient of variation as low as 0.079. In contrast, Mishan is situated on the edge of the Sanjiang Plain, characterized by relatively low and flat terrain with minimal obstruction to airflow. This makes its climate more susceptible to external air masses, resulting in greater interannual fluctuations in SCD (coefficient of variation: 0.254). Such differences highlight the complex interactions among climate patterns, topography, and snow cover dynamics. Trend analysis of snow cover duration shows that in Mohe, the slope is −0.3009 with a *p*-value of 0.0064, indicating a statistically significant decreasing trend. In Mishan, the slope is −0.1278 with a *p*-value of 0.5786, suggesting a non-significant decreasing trend. Both Mohe and Mishan are located within China’s major stable snow cover regions. Previous studies have demonstrated that under the background of global warming, snow cover duration in these areas has generally been decreasing [36,37].

### 2.3. Snow Physical Parameters

The physical parameters of snow directly affect the spatial distribution, temporal evolution, and observational accuracy of snow depth. Parameters such as snow density, snow water equivalent, snow grain size, and snow layer temperature collectively govern snow accumulation and ablation. In particular, snow grain size and density influence the snow albedo, thereby altering the energy balance and ultimately controlling the melt rate and snow depth variation [38]. A significant relationship exists between snow depth and snow albedo: when snow cover is thin, the underlying surface (e.g., soil and vegetation) absorbs part of the visible radiation transmitted through the snowpack, leading to a reduction in surface albedo. As snow depth increases, albedo gradually rises until a critical depth is reached [39]. The ERA5-Land Reanalysis (ELR) climate time series has proven valuable in meteorological research. As the land component of ERA5, it provides a spatial resolution of 0.1° × 0.1°. This dataset is generated based on the H-TESSEL land surface process model without coupling with the atmospheric module and without implementing a data assimilation process [40]. It should be noted that ELR employs data assimilation techniques—statistical methods that integrate multi-source observations (such as satellite remote sensing, radio soundings, surface meteorological stations, and numerical forecast outputs). Owing to its finer grid and specially optimized land surface model, ELR can better capture the influence of topography and surface heterogeneity on hydrological processes [41,42]. In this study, snow density and snow albedo were extracted from the ELR dataset as input parameters for the model, aiming to represent the spatiotemporal heterogeneity of snow characteristics in the process of snow depth inversion.

## 3. Methodology

**Figure 4:**
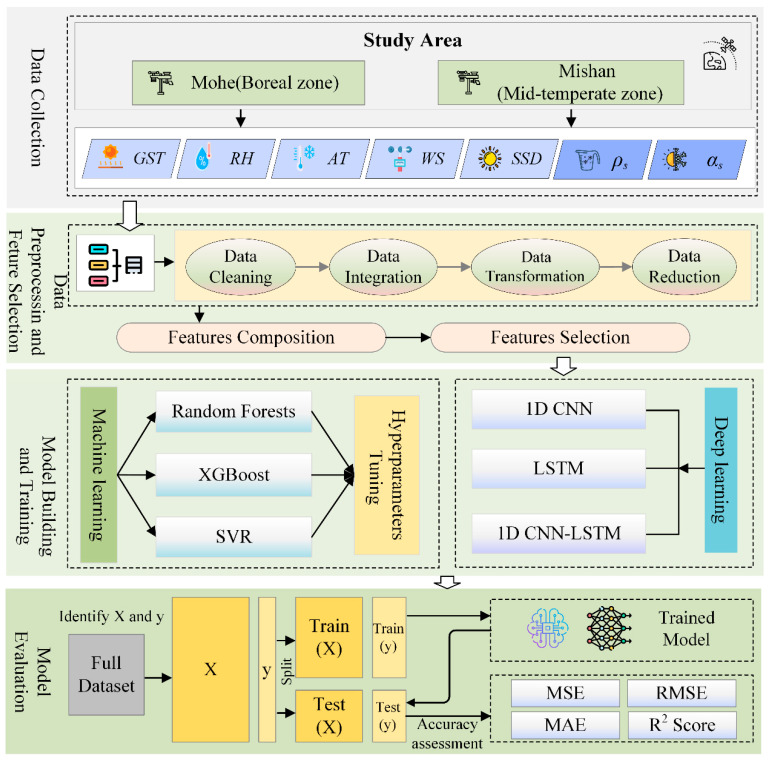
Flow chart of snow depth Prediction.

This study aims to establish a framework for daily snow depth prediction based on machine learning and deep learning, focusing on two typical snow-covered regions in China—Mohe and Mishan. The goal is to develop an end-to-end prediction workflow that spans from data collection to model evaluation. Figure 4 illustrates the workflow of the proposed method, outlining its four key steps in a flowchart format.

Step 1: Data Collection

Long-term daily meteorological and snow data from the Mohe and Mishan stations are selected, including ground temperature, relative humidity, air temperature, wind speed, sunshine duration, and corresponding measured snow depth records. A spatiotemporally representative modeling dataset is constructed from these observations.

Step 2: Data Preprocessing and Feature Selection

Raw data undergo quality control, including missing-value imputation, outlier detection and handling, and standardization to improve training efficiency. Correlation analysis and feature importance evaluation are then conducted to identify predictors that significantly influence snow depth variation, enhancing both the physical interpretability and predictive power of the input variables.

Step 3: Model Construction and Training

Both machine learning algorithms and deep learning models are employed. Using historical meteorological sequences as input and daily snow depth as output, the models are trained and hyper-parameter-tuned to capture the nonlinear dynamics underlying snow accumulation and ablation processes.

Step 4: Model Evaluation

Based on an independent validation set, model performance is compared in terms of prediction accuracy, stability, and generalization capability. Quantitative metrics such as Root Mean Square Error (RMSE) and Mean Absolute Error (MAE) are used for assessment. The most suitable machine learning and deep learning algorithms for accurate snow depth prediction are finally selected, providing a reliable tool for snow-cover dynamics simulation and snow-water resource management.

### 3.1. Feature Selection

**Figure 5:**
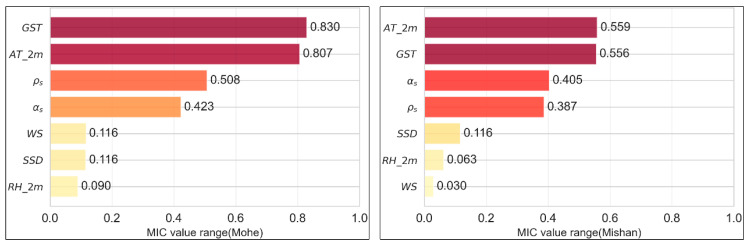
MIC values between each feature and snow depth.

The Maximal Information Coefficient (MIC) is a statistical measure used to quantify the strength of association between two variables, grounded in the concept of mutual information from information theory [42]. Unlike the Pearson correlation coefficient, which captures only linear relationships, MIC excels in detecting diverse and complex relational patterns, including linear, nonlinear, periodic, and other functional forms. The computation of MIC involves dynamically partitioning the data space through grid exploration to estimate the mutual information distribution between variables, with the maximal mutual information value normalized to the [0, 1] interval: a value close to 1 indicates a strong association between the variables, while a value near 0 suggests statistical independence. This characteristic makes MIC a powerful tool for feature selection and exploratory data analysis in uncovering deep variable relationships. Figure 5 presents the MIC calculation results for the two datasets. It can be observed that ground surface temperature (GST) and air temperature (AT) exhibit strong statistical dependencies with snow depth (MIC values of 0.830 and 0.807, respectively), indicating that thermodynamic processes constitute the primary mechanism influencing snow depth. In contrast, wind speed (WS), sunshine duration (SSD), and relative humidity (RH) show minimal influence (all with MIC values below 0.12), suggesting their limited direct impact on snow depth variation.

In time series analysis, the first-order lag is a method describing the relationship between the current value and its immediately preceding value. Meanwhile, within a data-driven framework, input features form the basis for the model to understand system behavior, and the way they are combined directly affects the depth of exploration into underlying mechanisms. In this study, we designed four feature combination schemes to evaluate the contribution of different variables to snow depth prediction, with detailed classification of predictive variables summarized in Table 2. Combination 1 employs the first-order lag values of five meteorological elements: ground temperature, air temperature, relative humidity, wind speed, and sunshine duration. Combination 2 adds the first-order lag value of snow depth to Combination 1. Combination 3 adds observed snow density and snow albedo data to Combination 1. Combination 4 consists of Combination 3 together with the first-order lag value of snow depth, i.e., it includes all feature variables.

### 3.2. Dataset Splitting

To construct and evaluate the snow depth simulation model, the historical dataset must be divided into a training set for model training and a test set for independently assessing model performance. This study adopts a fixed temporal split-point approach, with 1 August 1996, designated as the split point. The rationale for this choice is outlined below:

(1) Consideration of hydrological year completeness: Snow depth variation exhibits strong seasonality, and a complete snow accumulation–ablation cycle is critical for accurate simulation. Adopting the hydrological year (in this study, defined as 1 August to 31 July of the following year) as the analytical unit ensures that each data sample encompasses a full snow season. This approach also prevents data from a single snow season from being split across different sets, thereby preserving the physical process independence of both the training and test sets.

(2) Adherence to time series modeling rigor: To prevent future information leakage and ensure the fairness of model evaluation, it is essential that the test set chronologically follows the training set [43]. The fixed temporal split method effectively satisfies this prerequisite. In this study, the training set covers the period from 1 August 1961, to 31 July 1996, while the test set spans 1 August 1996, to 31 July 2016. These two periods are temporally continuous and non-overlapping, ensuring that the model is trained on “past” data and applied to predict “future” periods.

(3) Reasonable allocation of data volume: With this division, the sample size ratio between the training set and the test set is approximately 64%: 36% (about 35 years for training and 20 years for testing). This proportion is common in machine learning practice, as it ensures sufficient data for the model to learn complex patterns during training while providing an adequately long independent observation period for robust evaluation of model performance.

### 3.3. Machine Learning Models

#### 3.3.1. Random Forests

Random Forest Regression (RFR) is a powerful data-driven model based on ensemble learning [44]. This model effectively captures complex nonlinear relationships by constructing a large number of decision trees and aggregating their predictions, while also demonstrating excellent resistance to overfitting and strong robustness. Its core principle lies in generating a different training set for each tree through bootstrap sampling and introducing randomness during node splitting—only a random subset of features is considered when searching for the optimal split point. This dual randomness ensures diversity among the trees, thereby enhancing the overall generalization performance. For regression tasks, the final prediction of the model is the average of the outputs of all decision trees. Assuming the random forest consists of *B* decision trees, its prediction function can be defined as follows:

(1) $${\hat{f}}_{RF}\left(x\right)=\frac{1}{B}\sum_{b=1}^{B}T_{b}\left(x\right)$$

where $T_{b}(x)$ is the prediction of the *b*-th tree for sample *x*. The model is widely used in environmental modeling, hydrological forecasting, and related fields due to its ability to handle high-dimensional data and its built-in feature importance evaluation.

#### 3.3.2. XGBoost

XGBoost Regression (eXtreme Gradient Boosting) is an advanced gradient-boosted decision tree model and has become a leading algorithm in the machine learning field due to its outstanding predictive accuracy and computational efficiency [45]. Unlike the “bagging” approach used in Random Forest, XGBoost adopts the core idea of “boosting,” where a sequence of decision trees is built serially, with each subsequent tree focusing on correcting the errors made by the previous ones.

The core design of this algorithm lies in using a second-order Taylor expansion to rapidly and accurately optimize the loss function. Additionally, a regularization term is incorporated into the objective function to effectively mitigate overfitting, while computational efficiency aspects such as parallel processing and sparse data handling are deeply optimized. For a regression problem, given a dataset $D=\{(x_{i},y_{i})\}(|D|=n,x_{i}\in R^{m},y_{i}\in R)$, the XGBoost ensemble model consists of K additive trees. Its prediction model is the sum of the predictions from multiple tree models:

(2) $${\hat{y}}_{i}=\phi \left(x_{i}\right)=\sum_{k=1}^{K}f_{k}\left(x_{i}\right),f_{k}\in F$$

where ${\hat{y}}_{i}$ is the predicted value for the *i*-th sample, *f_k_* represents the *k*-th independent decision tree, and F denotes the function space composed of all possible decision trees. The model’s objective function consists of two parts: a loss function l and a regularization term Ω.

(3) $$L\left(\phi \right)=\sum_{i}l\left({\hat{y}}_{i},y_{i}\right)+\sum_{k}\Omega \left(f_{k}\right)$$

(4) $$\Omega \left(f\right)=\gamma T+\frac{1}{2}\lambda ||w{||}^{2}$$

where *T* is the number of leaf nodes in the tree, *w* is the weight (i.e., prediction score) of the leaf nodes, and *γ* and *λ* are regularization parameters that control model complexity. XGBoost minimizes the objective function through a scalable tree-boosting algorithm, which enables it to deliver outstanding performance in various regression prediction tasks.

#### 3.3.3. Support Vector Regression

Support Vector Machine Regression (SVR) is an extension of the Support Vector Machine (SVM) to regression problems. Its core idea is to find a regression function such that most training samples lie within an interval band (defined by the parameter ε) centered on this function [46]. By minimizing model complexity while allowing small-range errors, SVR achieves a robust model with strong generalization capability.

For nonlinear problems, SVR employs the kernel trick to map input data into a high-dimensional feature space, where linear regression is then performed. The core regression function is defined as:

(5) $$f\left(x\right)=W^{T}\phi \left(x\right)+b$$

where W is the weight vector, *b* is the bias term, and ϕ(x) is a nonlinear transformation function that maps the input data to a high-dimensional feature space. The optimization objective of this model simultaneously balances the flatness of the model (by minimizing ∥W∥) and tolerance to deviations from the training data, which is typically achieved by introducing slack variables and a trade-off parameter C.

### 3.4. Deep Learning Models

The formation and evolution of snow depth are essentially complex processes driven by meteorological factors involving long-term, multivariate coupling. This study leverages high-temporal-resolution and long-timespan meteorological monitoring data, which provide a rich information base for capturing the dynamic patterns of these processes. The core advantage of deep learning models lies in their ability to operate in an “end-to-end” manner, automatically learning effective feature representations and complex dependencies directly from vast amounts of raw time-series data, thereby maximizing the value of the data. To systematically evaluate the effectiveness of such methods in snow depth prediction, this study will select and compare three advanced architectures specifically designed for processing sequential data: the 1D CNN, which excels at local feature extraction; the LSTM, which captures long-term temporal dependencies; and the hybrid 1D CNN-LSTM model, which combines the strengths of both.

#### 3.4.1. 1D Convolutional Neural Network

The 1D Convolutional Neural Network (1D CNN) is a deep learning model specifically designed for processing sequential data and is widely applied in fields such as time series analysis [47]. This model can automatically and efficiently mine deep spatiotemporal features from data, achieving a good balance among accuracy, generalizability, and computational efficiency. As a result, it has become a commonly used tool for sequence processing. Its core computation is the one-dimensional convolution, which extracts local features through a sliding local filter. The formula is as follows:

(6) $$Y\left[i\right]=\sum_{j=0}^{k-1}X\left[i+j\right]\cdot W\left[j\right]+b$$

In the formula, *Y*[*i*] represents the value of the output feature map at position *i*; *X* denotes the input sequence, with *X*[*i*+*j*] being the element at position *i*+*j* of the input; *W* is the convolutional kernel weight vector, where *W*[*j*] represents the weight at position *j*; *k* is the kernel size, determining the width of the local receptive field; *b* is the learnable bias term; and the index *i* traverses all valid positions according to the stride. Through autonomous learning of its weight parameters, this local filter can automatically and efficiently identify key local patterns related to snow accumulation and ablation from the raw meteorological time series. It captures both short-term fluctuations and long-term trends in meteorological variables, thereby contributing to more accurate snow depth predictions in this study.

#### 3.4.2. Long Short-Term Memory (LSTM)

The Long Short-Term Memory (LSTM) network is a variant of RNN designed to address the gradient vanishing or explosion issues encountered during the training of traditional RNNs on long sequences [48]. Its core innovation lies in the introduction of a “cell state” as a long-term memory unit that persists throughout the sequence, along with three gating mechanisms—the forget gate, input gate, and output gate—that regulate the flow of information. Each gate employs a Sigmoid activation function, producing outputs between 0 and 1, corresponding to “complete forgetting” and “complete retention,” respectively.

The forget gate determines the information to be discarded from the cell state $C_{t-1}$ of the previous time step:

(7) $$f_{t}=\sigma \left(W_{f}\cdot \left[h_{t-1},x_{t}\right]+b_{f}\right)$$

The input gate is responsible for incorporating important information from the current time step into the cell state. It consists of the following operations:

(8) $$i_{t}=\sigma \left(W_{i}\cdot \left[h_{t-1},x_{t}\right]+b_{i}\right)$$

(9) $${\tilde{C}}_{t}=\tanh\left(W_{C}\cdot \left[h_{t-1},x_{t}\right]+b_{C}\right)$$

In the equations, *i_t_* controls the proportion of the candidate information ${\tilde{C}}_{t}$ to be retained, while ${\tilde{C}}_{t}$ contains the potential new information at the current time step.

The output gate generates the current hidden state *h_t_* based on the updated cell state $C_{t}$:

(10) $$o_{t}=\sigma \left(W_{o}\cdot \left[h_{t-1},x_{t}\right]+b_{o}\right)$$

(11) $$h_{t}=o_{t}⊙\tanh\left(C_{t}\right)$$

It serves as the output for the current time step and is passed to the next time step for prediction.

In snow depth prediction, the LSTM model can autonomously learn long-term dynamic patterns from meteorological sequences. By leveraging its gating mechanisms, it effectively retains critical information while filtering out irrelevant short-term fluctuations, thereby adapting to changing meteorological conditions and enhancing prediction accuracy.

#### 3.4.3. 1D CNN-LSTM

1D CNN-LSTM is a hybrid model that combines 1D CNN and LSTM, designed for processing one-dimensional sequential data. It effectively extracts local features while capturing long-term dependencies [49]. Its collaborative workflow can be summarized as follows:

(12) $$H_{\mathrm{cnn}}=\mathrm{CNN}\left(X_{\mathrm{raw}}\right)$$

In this formulation, $H_{\mathrm{cnn}}\in R^{T^{\prime }\times F}$ represents the high-level feature sequence output by the CNN layer, where *T*′ denotes the new sequence length after convolution and pooling *T*′ < *T*, and *F* is the number of filters. The LSTM processes *H*_cnn_ as a time series step by step at each time step *t*′, with the input being $h_{\mathrm{cnn},t^{\prime }}\in R^{F}$. The final hidden state $h_{T^{\prime }}$ captures the long-term temporal dependencies among the features.

This type of model compresses the raw sequence into compact feature sequences via CNN, and then LSTM captures the temporal evolution patterns of these features. In complex time-series regression tasks, it typically outperforms standalone CNN or LSTM models [50].

In this study, the constructed 1D CNN-LSTM hybrid model accepts input sequences with a time step of one day and 5 to 8 features. The data were first standardized using Z-score normalization based on the training set. The model adopts a minimal CNN architecture, consisting of only one Conv1D layer with 32 filters of size 1, followed by a Dropout layer with a rate of 0.3. This is then connected to a two-layer LSTM structure. The first LSTM layer contains 50 units, and the second LSTM layer contains 25 units, with a Dropout rate of 0.2 applied after each LSTM layer. Finally, the output is generated through three fully connected layers (with 20, 10, and 1 neurons, respectively; the first two layers use ReLU activation, and the output layer uses linear activation). The model was trained using the Adam optimizer (learning rate 0.001) for 100 epochs with a batch size of 16, and the loss function was mean squared error. Under this configuration, the model contains a total of 25,229 trainable parameters.

### 3.5. Model Evaluation

In the regression task of snow depth prediction, we employ four classic evaluation metrics to comprehensively assess the predictive performance of the model: Root Mean Square Error (RMSE), Mean Absolute Error (MAE), Mean Squared Error (MSE), and the Coefficient of Determination (R^2^). These metrics reflect the degree of difference between the model’s predicted values and the actual observed values from different perspectives.

RMSE is the standard deviation of prediction errors. By squaring the errors, it imposes a higher penalty on larger errors, making it sensitive to outliers in the predictions. The value of RMSE ranges from [0, +∞), and a smaller value indicates higher prediction accuracy of the model. In the formula, ${SD}_{i}$ represents the true snow depth of the *i*-th sample, ${\hat{SD}}_{i}$ denotes the corresponding predicted value, and n is the number of samples.

(13) $$\mathrm{RMSE}=\sqrt{\frac{1}{n}\sum_{i=1}^{n}({SD}_{i}-{\hat{SD}}_{i}{)}^{2}}$$

MAE calculates the average of the absolute differences between predicted and actual values. It is less sensitive to outliers compared to RMSE, providing a more robust measure of the model’s average prediction error. The value of MAE also ranges from [0, +∞), with smaller values indicating better model performance.

(14) $$\mathrm{MAE}=\frac{1}{n}\sum_{i=1}^{n}|{SD}_{i}-{\hat{SD}}_{i}|$$

MSE is the average of the squared differences between predicted and actual values. As the foundational calculation step for RMSE, it similarly assigns greater weight to larger errors. The value of MSE ranges from [0, +∞), with smaller values indicating more accurate predictions by the model.

(15) $$\mathrm{MSE}=\frac{1}{n}\sum_{i=1}^{n}({SD}_{i}-{\hat{SD}}_{i}{)}^{2}$$

R^2^ reflects the proportion of variance in the target variable explained by the model, measuring the goodness-of-fit between predicted values and actual values. The value of R^2^ ranges from (−∞, 1]. A value closer to 1 indicates a stronger explanatory power of the model. When R^2^ = 1, it represents a perfect prediction; R^2^ = 0 indicates that the model performs only as well as the mean prediction; while a negative value suggests that the model performs worse than the simple mean baseline.

(16) $$R^{2}=1-\frac{\sum_{i=1}^{n}(SD-{\hat{SD}}_{i}{)}^{2}}{\sum_{i=1}^{n}({SD}_{i}-\overset{-}{SD}{)}^{2}}$$

To comprehensively compare the performance of different models and address potential conflicting conclusions drawn by different evaluation metrics, this study adopts a two-stage integrated judgment strategy.

Stage 1: Comprehensive Score Calculation

First, to eliminate the influence of different measurement units across metrics, the RMSE and R^2^ values of each model on the test set are converted into their respective ranking values (Rank) among all candidate models. Subsequently, a weighted comprehensive score is calculated as follows:

(17) $$\mathrm{Score}=0.6\times \mathrm{Rank}\left(\mathrm{RMSE}\right)+0.4\times \mathrm{Rank}\left(R^{2}\right)$$

where RMSE is assigned a higher weight (0.6) than *R*^2^ (0.4), reflecting the priority of minimizing absolute prediction errors over maximizing explained variance in snow depth prediction applications. The model with the lowest comprehensive score is identified as the preliminary optimal model.

Stage 2: Robustness Diagnosis of Error Distribution

When multiple models exhibit close comprehensive scores or when the preliminary optimal model requires robustness verification, the following analyses are introduced:

- Comparison of Mean Absolute Error (MAE): As a direct measure of average error magnitude, MAE is used to confirm trends reflected by RMSE.
- Analysis of Error Distribution Dispersion: The difference between RMSE and MAE (RMSE–MAE) is calculated. This difference is sensitive to large errors (outliers) in predictions. A larger difference indicates a heavy-tailed error distribution, meaning the model may produce a small number of severe prediction errors, which is undesirable in practical applications.

The final model selection follows these principles: Priority is given to the model with the lowest comprehensive score; if scores are similar, preference is given to the model with a lower MAE and a smaller (RMSE–MAE) difference.

## 4. Results

To conduct daily snow depth prediction research, this paper systematically compares the predictive performance of traditional machine learning models and deep learning models at two meteorological monitoring stations—Mohe and Mishan. Using both univariate and multivariate input modes, and through multi-feature combination simulation experiments, it focuses on analyzing the impact of ground observation data from different periods in the two regions on prediction accuracy.

### 4.1. Snow Depth Inversion Results Using Machine Learning

In this section, we compare three distinct machine learning algorithms: RF, XGBoost, and SVR, which belong to different categories of machine learning methods—tree-based methods (RF, XGBoost) and kernel-based methods (SVR). Models for both regions are configured with a unified hyperparameter optimization setup.

As shown in Table 3, in the Mohe region, all models performed excellently with the C2 and C4 combinations. Both on the training and test sets, R^2^ values were generally above 0.90, while MSE, MAE, and RMSE values remained low. These two combinations both include the *SD_t-_*_1_ feature variable. In contrast, all machine learning models based on the C1 and C3 combinations demonstrated relatively poor generalization ability: training set R^2^ ranged from 0.45 to 0.80, while test set R^2^ dropped significantly (as low as 0.01), indicating clear overfitting. The MSE and RMSE on the test set increased notably, suggesting poor predictive performance on unseen data. This demonstrates that *SD_t-_*_1_ is the most important input variable for prediction. Among the models, XGBoost based on C4 performed best during testing, achieving an R^2^ of 0.9890 and an RMSE of 1.19 cm. RF based on C4 ranked second, with an R^2^ of 0.9872 and an RMSE of 1.29 cm. SVR also performed well, reaching an R^2^ of 0.9642 and an RMSE of 2.17 cm when using all feature variables.

At the Mohe station, the XGBoost model demonstrated excellent generalization capability under the C2 and C4 combinations, without showing significant overfitting. For the C2 combination, the training set R^2^ was 0.9623, and the test set R^2^ was 0.9539, representing a decrease of only 0.84%. The RMSE slightly increased from 0.68 in the training set to 1.29 in the test set, a relative increase of 90%. However, given the small baseline value, the absolute increment of 0.61 remains within a reasonable range. The performance under the C4 combination was even more remarkable, with the training set R^2^ reaching as high as 0.9924 and the test set maintaining an excellent level of 0.9890, a decrease of only 0.34%. The RMSE increased from 0.76 to 1.19, a growth of 57%, with an absolute increment of 0.43. For both combinations, the key performance indicators showed high consistency between the training and test sets, with R^2^ decreases of less than 1%. This indicates that the model not only effectively learned the patterns in the training data but also generalized well to unseen data.

**Figure 6:**
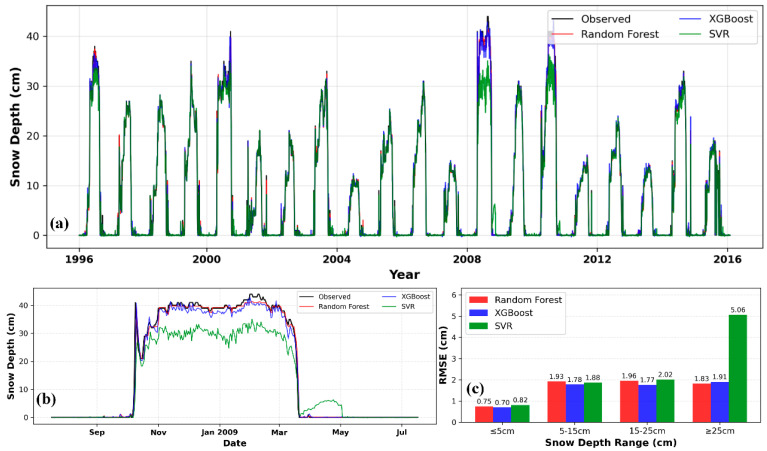
Daily snow depth prediction in Mohe using machine learning. (**a**) Snow depth prediction curve; (**b**) Snow depth prediction curve for the 2009–2010 hydrological year; (**c**) Comparison of model RMSE under different snow depth conditions.

**Figure 7:**
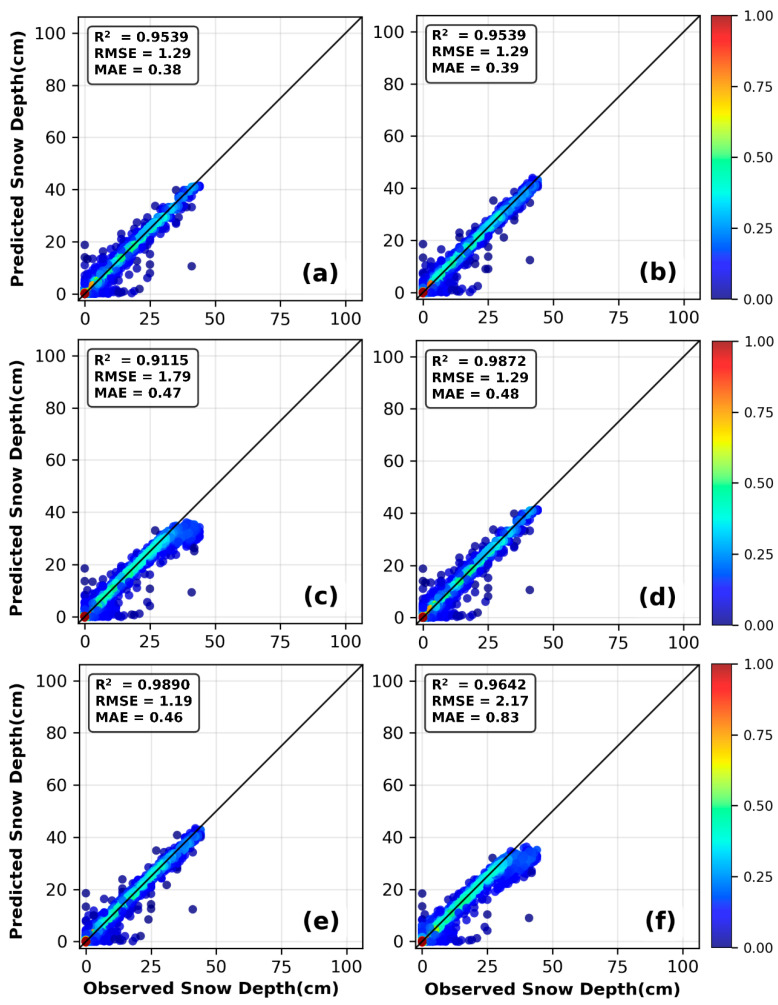
Comparison of observed values and predicted values by RF, XGBoost, and SVR models in Mohe: (**a**–**c**) are based on the C2 feature combination, and (**d**–**f**) are based on the C4 feature combination.

Figure 6a shows a comparison between the daily snow depth predictions and observed values for the RF, XGBoost, and SVR under the C4 combination. All three models successfully captured the overall trend of snow depth variation. From Figure 6b,c, it can be observed that as snow depth increases, the performance of each model fluctuates to varying degrees: In the shallow/medium snow depth ranges (≤5 cm, 5–15 cm, and 15–25 cm), the RMSE values of the three models are all at a relatively low level and close to each other, with XGBoost achieving the best performance (RMSE = 0.70 cm). However, in the deep snow depth range (≥25 cm), the model performance diverges significantly: SVR has the worst accuracy with an RMSE of 5.96 cm, while RF (1.61 cm) and XGBoost (1.91 cm) have significantly lower errors. Among them, RF is the model with the best accuracy in deep snow scenarios. Figure 7 presents scatter plots of observed snow depth versus snow depth inverted by machine learning models under the C2 and C4 combinations at the Mohe meteorological station during the 1996–2016 hydrological years. All scatter plots display a clear positive correlation trend, with data points densely clustered along a line from the bottom left to the top right. The majority of points closely surround the regression line. These results consistently indicate that all three machine learning models achieved excellent fitting performance under the C2 and C4 combinations.

For the Mishan region, the XGBoost model based on the C4 combination performed best (see Table 4), achieving an R^2^ of 0.9549, while its MSE (1.65), MAE (0.39), and RMSE (1.28) were the lowest among all model combinations, demonstrating the highest prediction accuracy and stability. Random Forest (C2 combination) and XGBoost (C2 combination) also performed well on the test set, both reaching an R^2^ of 0.9539, very close to the optimal model. The C2 and C4 combinations significantly improved the prediction performance of all models, with test-set R^2^ values all above 0.9.

At the Mishan station, the XGBoost model also demonstrated good generalization performance under the C2 and C4 combinations, with no significant signs of overfitting. For the C2 combination, the training set R^2^ was 0.9623, and the test set R^2^ was 0.9539, a decrease of 0.84%. The RMSE increased from 0.68 to 1.29, representing a 90% increase, with an absolute increment of 0.61. For the C4 combination, the training set R^2^ was 0.9700, and the test set R^2^ was 0.9549, a decrease of 1.55%. The RMSE increased from 0.60 to 1.28, a growth of 113%, with an absolute increment of 0.68. Although the relative percentage increases in RMSE may appear high, the absolute increments are all less than 0.7, and the test set RMSE values remain at a low level below 1.3. This indicates that the slight increase in error is more likely due to random fluctuations inherent in the data rather than model overfitting. More importantly, the test set R^2^ for both combinations maintained an excellent level above 0.95, with minimal performance differences compared to the training set, demonstrating that the model effectively captured the underlying patterns in the data.

**Figure 8:**
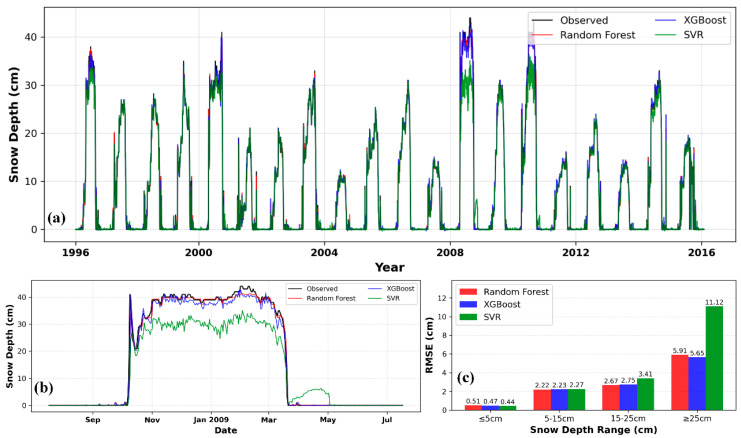
Daily snow depth prediction in Mishan (C4). (**a**) Snow depth prediction curve; (**b**) Snow depth prediction curve for the 2009–2010 hydrological year; (**c**) Comparison of model RMSE under different snow depth conditions.

**Figure 9:**
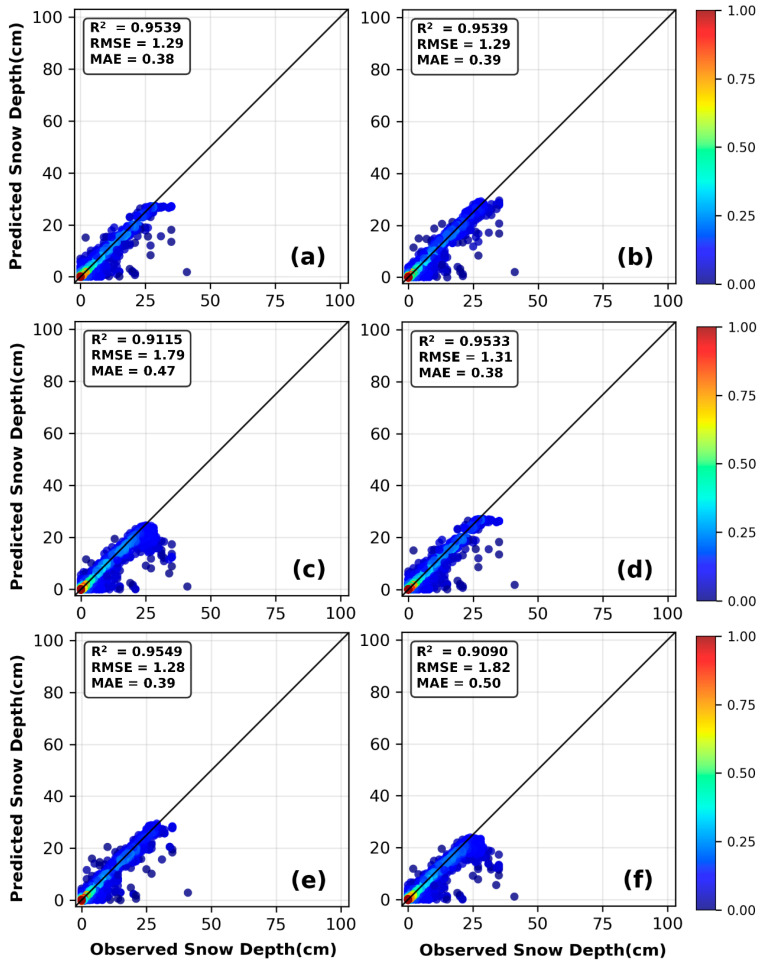
Comparison of observed values and predicted values by RF, XGBoost, and SVR models in Mishan: (**a**–**c**) are based on the C2 feature combination, and (**d**–**f**) are based on the C4 feature combination.

Through Figure 8a,b, we visualized the snow-depth predictions of the three machine-learning models using the C4 combination on the test set. In contrast, all models showed severe performance degradation under the C1 and C3 combinations. Especially for the SVR model under the C1 combination, the test-set R^2^ was only 0.0047, indicating that the model completely failed to capture the underlying patterns and suffered from serious overfitting or underfitting. This suggests that SVR is more sensitive to input combinations and exhibits lower robustness compared to RF and XGBoost. Figure 8c illustrates the performance variations in all machine learning models under different snow depth conditions. In the low snow depth range (≤5 cm), the RMSE of all three models is relatively low, with SVR performing the best (RMSE = 0.44 cm). In the 5–15 cm snow depth range, the RMSE of each model rises significantly: RF, XGBoost, and SVR reach 2.22 cm, 2.31 cm, and 2.27 cm respectively. When entering the 15–25 cm snow depth range, the performance of all models degrades further—RF still maintains the best performance (RMSE = 2.65 cm), followed by XGBoost (RMSE = 3.17 cm) and SVR (RMSE = 3.41 cm). In the snow depth range of ≥25 cm, the performance of all models drops sharply, with SVR’s RMSE reaching 11.12 cm. The scatter plots in Figure 9 show that all three machine-learning models could effectively capture the observed snow depth. It is worth noting that for RF and SVR, prediction accuracy under the C2 combination was higher than under C4, a result that highlights the critical role of feature engineering in machine learning.

### 4.2. Snow Depth Inversion Results Using Deep Learning

To systematically compare the performance of deep learning models in snow depth prediction for the Mohe and Mishan regions, this study selected three architectures for experimentation: 1D CNN, LSTM, and 1D CNN-LSTM. All models maintained strict consistency in network depth, hyperparameters, and training strategies in order to eliminate the impact of configuration differences on performance evaluation and ensure the comparability of the results.

As shown in Table 5, the statistical results of models at the Mohe station indicate that data combinations significantly impact model performance. Among the four combinations, C2 is the optimal fit for all models, and the 1D CNN stands out with the highest accuracy under this combination. Additionally, the 1D CNN maintains consistent advantages across the remaining combinations (C1, C3, C4), outperforming both the LSTM and 1D CNN-LSTM models overall. Specifically, in the training set of the C2 combination, the 1D CNN achieves the highest R^2^ (0.9868) and the lowest values for all error metrics—MSE (1.03), MAE (0.42), and RMSE (1.01)—which are notably superior to those of the LSTM (R^2^ = 0.9802, RMSE = 1.24) and 1D CNN-LSTM (R^2^ = 0.9812, RMSE = 1.21). In the testing set, the 1D CNN’s advantages are further highlighted: it delivers the highest R^2^ (0.9878) and the lowest MSE (1.59), MAE (0.57), and RMSE (1.26), while the LSTM (RMSE = 1.86) and 1D CNN-LSTM (RMSE = 1.60) exhibit higher error metrics. For the C1 and C3 combinations, all models demonstrate relatively poor overall performance; however, the 1D CNN still achieves substantially higher test-set R^2^ values (0.4595 for C1 and 0.5185 for C3) and significantly lower RMSE values (8.40 for C1 and 7.92 for C3) compared to the other two models, making it the only model with effective predictive capability under these two combinations. As the second-best combination, C4 sees the three models achieve similar R^2^ values in the training set, but the 1D CNN still performs marginally better in terms of error metrics. In the testing set, the 1D CNN maintains its leading position with an RMSE of 1.29 and an R^2^ of 0.9871, outperforming the LSTM (RMSE = 1.41) and 1D CNN-LSTM (RMSE = 2.05). In summary, the 1D CNN exhibits superior stability and predictive accuracy across all data combinations at the Mohe station, with the most prominent advantages under the optimal C2 combination, making it the preferred model for snow depth prediction at this station.

**Figure 10:**
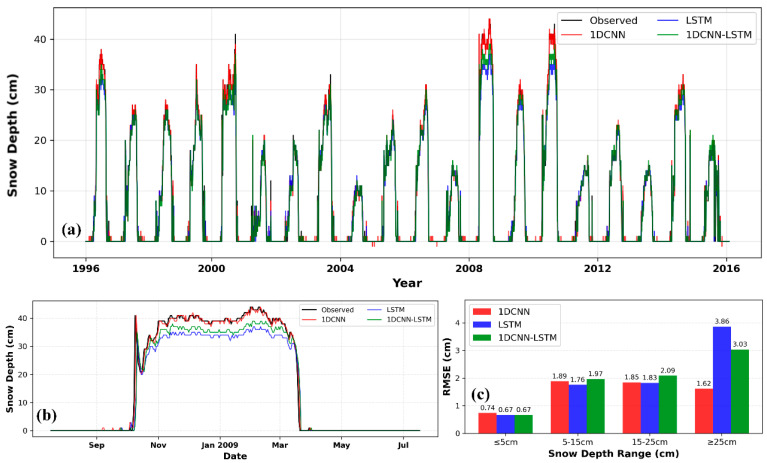
Daily snow depth prediction in Mohe using deep learning models (C2). (**a**) Snow depth prediction curve; (**b**) Snow depth prediction curve for the 2009–2010 hydrological year; (**c**) Comparison of model RMSE under different snow depth conditions.

From the curves in Figure 10a,b, it can be observed that all models are able to capture the overall variation trend of snow depth during the accumulation, stable, and ablation periods. However, the prediction curve of the 1D CNN closely matches the observed curve, and it exhibits the strongest ability to capture snow-depth peaks. LSTM and 1D CNN-LSTM show slight underestimation or delay at some peaks. Figure 10c illustrates the RMSE performance of three models—1D CNN, LSTM, and 1D CNN-LSTM—across different snow depth ranges. In the intervals of ≤5 cm, 5–15 cm, and 15–25 cm, all three models maintain relatively low RMSE values (0.67–2.03 cm), with comparable performance and good prediction accuracy. However, in the deep snow depth range (≥25 cm), significant performance divergence emerges: 1D CNN achieves the highest accuracy with an RMSE of 1.62 cm, followed by 1D CNN-LSTM (3.03 cm), while LSTM exhibits the lowest performance with an RMSE of 3.86 cm.

**Figure 11:**
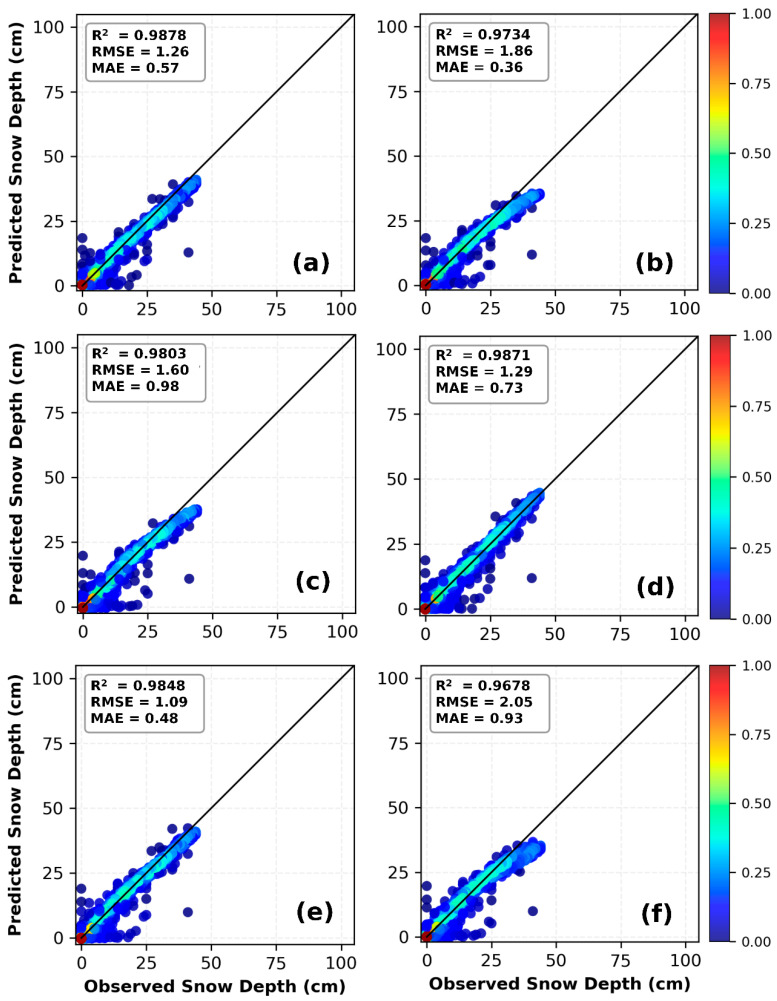
Comparison of observed values and predicted values by 1D CNN, LSTM, and 1D CNN-LSTM models in Mohe: (**a**–**c**) are based on the C2 feature combination, and (**d**–**f**) are based on the C4 feature combination.

Preliminary analysis suggests the following possible reasons: First, the feature combination that includes the first-order lag of snow depth already provides a strong predictive signal, thereby reducing the need to model complex long-term temporal dependencies and diminishing the value of the LSTM module. Second, the CNN front-end compresses or transforms the input sequence, resulting in the loss or distortion of certain local pattern information that is critical for prediction when it is passed to the LSTM. Third, the patterns and regularity in the data have not yet reached a level that requires such a complex architecture for interpretation; thus, increasing model complexity does not lead to performance gains. The scatter plots in Figure 11 illustrate the relationship between predicted and observed snow depths for the three deep-learning models under the C2 and C4 combinations. All models demonstrate high prediction accuracy, with scatter points tightly clustered around the 1:1 diagonal line, confirming that combining temporal snow-depth data with meteorological factors enhances model performance. The scatter plots in Figure 11e,f show relatively more pronounced dispersion, especially at greater snow depths, which is consistent with the statistical data presented in Table 6.

The prediction results at the Mishan station (see Table 6) indicate that combinations significantly influence model performance. Among all combinations, C2 is the optimal configuration for all models, and the 1D CNN demonstrates the highest accuracy under this combination. It also maintains stable competitiveness across the remaining combinations (C1, C3, C4), outperforming both the LSTM and 1D CNN-LSTM models overall. Specifically, in the training set of the C2 combination, the 1D CNN leads with comprehensive metrics: R^2^ = 0.9369, MSE = 0.77, MAE = 0.36, and RMSE = 0.88, which are notably superior to those of the LSTM (R^2^ = 0.9347, RMSE = 0.90) and 1D CNN-LSTM (R^2^ = 0.9282, RMSE = 0.94). In the testing set, the 1D CNN’s advantages are further consolidated, achieving the highest R^2^ = 0.9617 and the lowest values for all error metrics (MSE = 1.40, MAE = 0.40, RMSE = 1.18), while the LSTM (RMSE = 1.22) and 1D CNN-LSTM (RMSE = 1.34) exhibit sequentially higher errors. Under the C1 combination, all models show relatively poor overall performance; although the 1D CNN’s test-set R^2^ = 0.2128 is slightly lower than that of the LSTM, its RMSE = 5.37 is marginally different from the other two models, indicating comparable error control capability. In the C3 combination, the 1D CNN achieves the lowest training-set RMSE = 2.39, and its test-set RMSE = 4.23 is close to those of the other models, demonstrating good performance balance. As the second-best combination, C4 sees the 1D CNN and LSTM deliver nearly identical performance in the training set (both outperforming the 1D CNN-LSTM), while in the testing set, the 1D CNN’s R^2^ = 0.9471 and RMSE = 1.39 are slightly inferior to the LSTM but significantly better than the 1D CNN-LSTM. In summary, the 1D CNN performs exceptionally well under the core optimal C2 combination and exhibits strong stability and generalization across all combinations, making it the comprehensively optimal model for snow depth prediction at the Mishan station.

**Figure 12:**
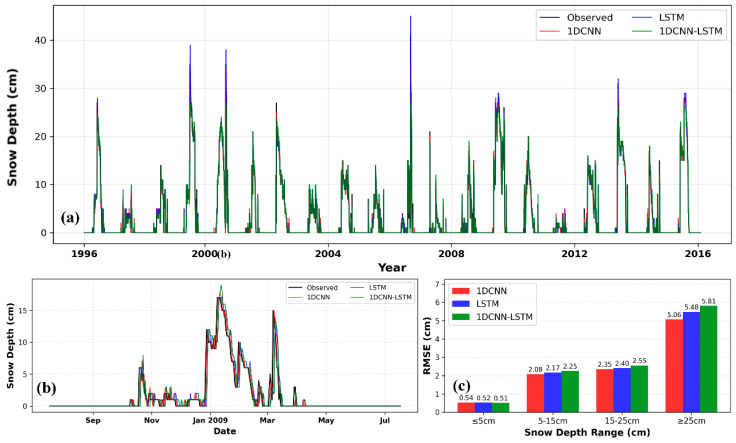
Daily snow depth prediction in Mishan using deep learning models (C2). (**a**) Snow depth prediction curve; (**b**) Snow depth prediction curve for the 2009–2010 hydrological year; (**c**) Comparison of model RMSE under different snow depth conditions.

Figure 12a visually illustrates the overall fit between the predicted and observed values from the deep learning models across the entire study period. All models successfully captured the core dynamics of interannual and seasonal snow cover variations. From Figure 12b, it can be seen that the 1D CNN’s prediction curve aligns best with the observed values, particularly during peak snow accumulation periods (e.g., around January 2009), where it tracks snow depth changes more accurately. In contrast, the LSTM model shows slight lagging or underestimation here. This advantage is confirmed by the quantitative evaluation in Figure 12c: In the shallow snow depth range (≤5 cm), all three models achieve relatively low RMSE values (0.51–0.52 cm), demonstrating good performance with minimal discrepancies. As snow depth increases (5–15 cm and 15–25 cm), the RMSE of the three models gradually rises but remains relatively close (2.06–2.55 cm), maintaining a generally high level of prediction accuracy. However, in the deep snow depth range (≥25 cm), significant performance divergence occurs: 1DCNN outperforms the other two models with an RMSE of 5.06 cm, which is lower than that of LSTM (5.48 cm) and 1DCNN-LSTM (5.51 cm), making it the most accurate model for deep snow scenarios.

**Figure 13:**
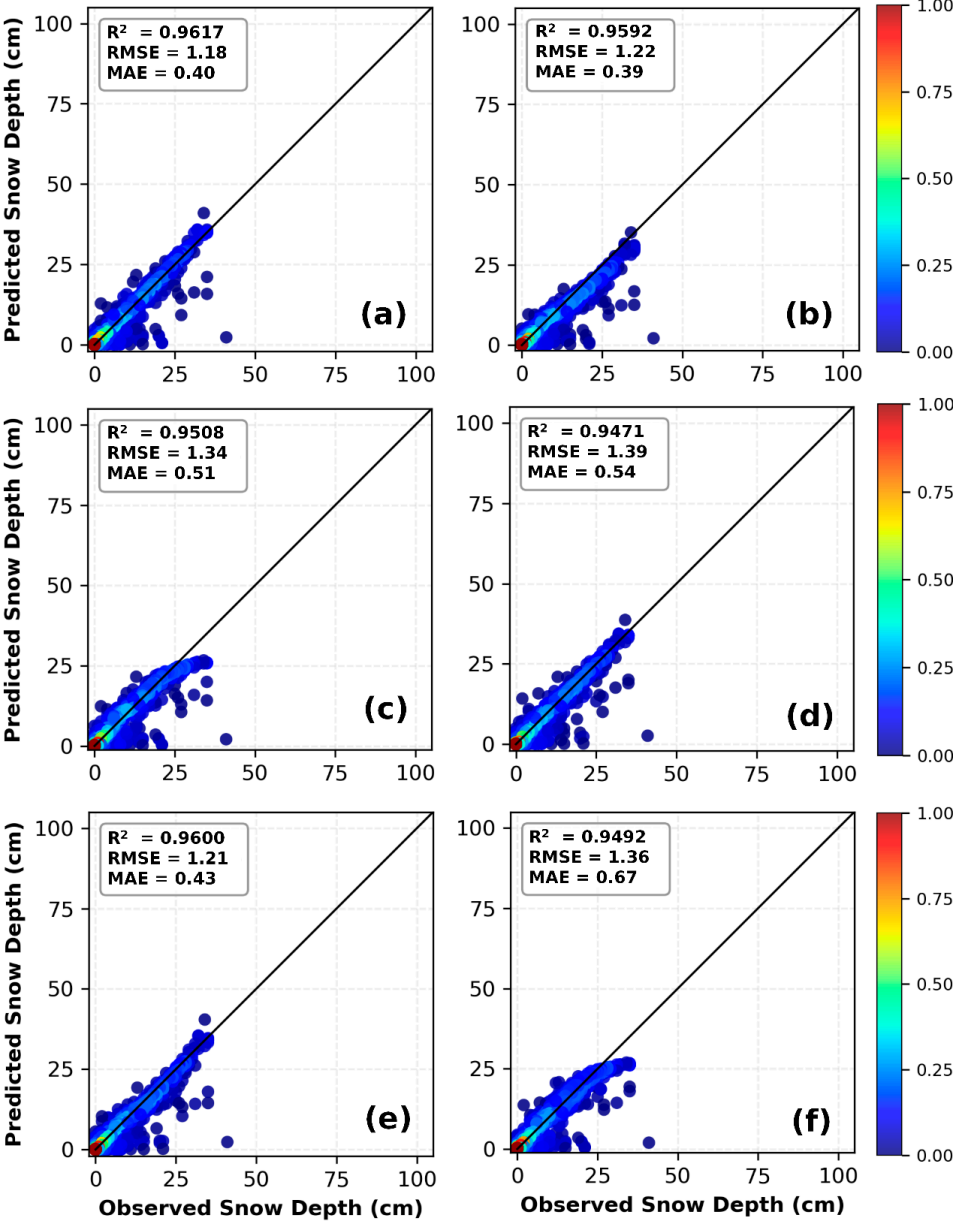
Comparison of observed values and predicted values by 1D CNN, LSTM, and 1D CNN-LSTM models in Mishan: (**a**–**c**) are based on the C2 feature combination, and (**d**–**f**) are based on the C4 feature combination.

Figure 13 compares the snow depth prediction performance of the 1D CNN, LSTM, and 1D CNN-LSTM models in the Mishan region under the C2 and C4 feature combinations. All scatter plots show a significant linear clustering trend, with data points tightly distributed near the diagonal line, visually confirming a very strong positive correlation between predicted and observed values. Among them, the point cloud in Figure 13a clusters most tightly and uniformly along the diagonal with fewer outliers, directly reflecting its optimal prediction accuracy. In terms of scatter concentration, the overall performance of the C2 combination is slightly superior to that of the C4 combination.

In summary, the two structurally simpler models, 1D CNN and LSTM, demonstrated powerful sequence modeling capabilities. The 1D CNN, leveraging its efficiency in local feature extraction, delivered the best performance with the C2 combination. Conversely, the LSTM exhibited greater robustness across multiple combinations due to its ability to capture long-term dependencies. In contrast, the more complex 1D CNN-LSTM hybrid model did not show the anticipated synergistic advantages; its performance under both C2 and C4 combinations was slightly inferior to that of the two simpler models. This suggests that model complexity needs to be appropriately matched with the characteristics of the data and the complexity of the problem at hand.

## 5. Discussion and Conclusions

This study systematically compares the performance of machine learning and deep learning models for daily snow depth inversion—by integrating meteorological factors and ERA5-Land snow-related physical parameters—based on long-term observational data from two representative stations (Mohe and Mishan) in Heilongjiang Province, China, spanning the hydrological years 1961 to 2015.

The experimental results consistently indicate that the first-order lag of snow depth is the most critical variable for predicting daily snow depth, a conclusion applicable to both machine learning and deep learning models. This phenomenon is universally observed at the Mohe and Mishan stations, which have distinct climatic and underlying surface conditions. It demonstrates that the high autocorrelation and persistence inherent to snow depth itself constitute the core physical mechanism governing its daily variations. Consequently, for sites with continuous observational records, establishing a prediction framework centered on historical snow depth data offers strong generalizability and robustness.

This study attempted to introduce snow density and snow albedo from the ERA5-Land reanalysis dataset, aiming to enhance the physical interpretability of the models. However, the results indicate that these parameters did not effectively improve model performance. Even when combined with the first-order lag of snow depth, the resulting gain in accuracy was relatively limited and was not consistently observed across all models and sites. This reflects a limitation of current data-driven models: while they excel at learning and leveraging strongly correlated signals, their ability to integrate and utilize auxiliary variables that are physically meaningful but weakly correlated with prediction targets remains constrained. This may be related to uncertainties inherent in the reanalysis data itself, the representativeness scale of the parameters, and the models’ inability to effectively capture the nonlinear coupling mechanisms between these parameters and snow depth variation. Future hybrid modeling efforts will require more sophisticated designs to achieve a deeper integration of physical mechanisms and data-driven statistical patterns.

From a model perspective, XGBoost and 1D CNN demonstrated superior performance. XGBoost showcased its excellent capability in handling complex nonlinear relationships, achieving an R^2^ of 0.989 and an RMSE of only 1.19 cm on the test set. The performance of 1D CNN was particularly impressive, outperforming all other deep learning models. Under the C2 combination at the Mohe station, it achieved an R^2^ of 0.9878 and an RMSE of 1.26 cm, while at the Mishan station under the same combination, it attained an R^2^ of 0.9617 and an RMSE of 1.18 cm. The strength of 1D CNN lies in its ability to automatically and efficiently extract local fluctuation patterns related to snow depth variation from raw meteorological sequences while maintaining relatively good robustness even in the absence of the first-order lagged snow depth value (C1 and C3 combinations). In contrast, LSTM, which is specifically designed for long time-series data, did not show an advantage in this study. Its highest accuracy on the test set was achieved under the C4 combination at the Mohe station, with an R^2^ of 0.9848 and an RMSE of 1.41 cm. The more complex 1D CNN-LSTM hybrid model also failed to achieve a performance breakthrough, with its highest accuracy on the test set being under the C2 combination at the Mohe station, yielding an R^2^ of 0.9803 and an RMSE of 1.60 cm. This indicates that model performance does not simply improve with increased complexity; its effectiveness fundamentally depends on how well it aligns with the characteristics of the problem being addressed.

This study has achieved acceptable results. Future work could focus on the following aspects: (1) exploring more in-depth physics-informed machine learning hybrid modeling, for example by embedding simplified physical processes from snowpack models as constraints or prior knowledge; (2) integrating multi-source remote-sensing data (optical, passive microwave, SAR), reanalysis products, and elevation information to build spatiotemporal fusion models for large-scale, continuous snow-depth dynamic monitoring and mapping; (3) investigating more advanced temporal network architectures to more effectively capture potential long-range and multi-scale dependencies between meteorological drivers and snow-depth response; (4) extending this framework to the regional scale by utilizing spatially continuous reanalysis data and remote sensing data to drive the model, and systematically assessing its spatial transferability and generalization capacity through the design of rigorous cross-regional validation schemes.

## Figures and Tables

**Table 1:** Statistics on Differences in Temperature, Humidity, and Snow Depth between Mohe and Mishan from Long-Term Observations.

Meteorological Variable	Units	Weather Station	Samples	Mean	Min	Max	Standard Deviation
Relative Humidity (2 m)	%	Mohe	20,089	69.45	15	100	13.06
		Mishan		66.99	12	100	15.21
Air Temperature (2 m)	°C	Mohe	20,089	−4.09	−46.7	28.1	17.95
		Mishan		3.89	−29.8	29.9	14.21
Snow Depth	cm	Mohe	20,089	7.08	0	53	9.88
		Mishan		1.96	0	41	9.3

**Table 2:** Feature Combination Design.

Variable Combinations	Variables Included
C1	*GST_t-_*_1_, *AT_t-_*_1_*, RH_t-_*_1_, *WS_t-_*_1_, *SDD_t-_*_1_
C2	*GST_t-_*_1_, *AT_t-_*_1_*, RH_t-_*_1_, *WS_t-_*_1_, *SDD_t-_*_1_, *SD_t-_*_1_
C3	*GST_t-_*_1_, *AT_t-_*_1_*, RH_t-_*_1_, *WS_t-_*_1_, *SDD_t-_*_1_*, ρ_s_*_,*t*-1_, α*_s_*_,*t*-1_
C4	*GST_t-_*_1_, *AT_t-_*_1_*, RH_t-_*_1_, *WS_t-_*_1_, *SDD_t-_*_1_*, ρ_s_*_,*t*-1_, α*_s_*_,*t*-1_, *SD_t-_*_1_

**Table 3:** Statistics on snow depth prediction performance of machine learning in Mohe.

Station	ML Models	Combinations	Training Set				Testing Set			
			R^2^	MSE	MAE	RMSE	R^2^	MSE	MAE	RMSE
Mohe	RF	C1	0.7056	23.01	2.76	4.79	0.3539	84.35	4.73	9.18
		C2	0.9353	0.89	0.27	0.79	0.9539	1.68	0.38	1.29
		C3	0.7257	21.43	2.65	4.63	0.4533	71.38	4.44	8.44
		C4	0.9850	1.17	0.40	1.08	0.9872	1.67	0.48	1.29
	XGBoost	C1	0.7723	17.79	2.39	4.21	0.0104	129.20	5.97	11.36
		C2	0.9623	0.46	0.22	0.68	0.9539	1.68	0.39	1.29
		C3	0.4533	71.38	4.44	8.45	0.1425	111.94	5.67	10.58
		C4	0.9924	0.59	0.30	0.76	0.9890	1.43	0.46	1.19
	SVR	C1	0.6694	25.83	2.85	5.08	0.1916	105.53	5.42	10.27
		C2	0.9201	0.98	0.26	0.99	0.9115	3.23	0.47	1.79
		C3	0.6891	24.30	2.78	4.92	0.3322	87.19	5.08	9.33
		C4	0.9816	1.43	0.40	1.19	0.9642	4.67	0.83	2.17

**Table 4:** Statistics on snow depth prediction performance of machine learning in Mishan.

Station	ML Models	Combinations	Training Set				Testing Set			
			R^2^	MSE	MAE	RMSE	R^2^	MSE	MAE	RMSE
Mishan	RF	C1	0.4491	6.77	1.14	2.60	0.2332	28.04	2.20	5.29
		C2	0.9353	0.79	0.27	0.89	0.9539	1.68	0.38	1.29
		C3	0.6134	4.75	0.96	2.18	0.3985	22.00	1.89	4.69
		C4	0.9361	0.78	0.27	0.88	0.9533	1.70	0.38	1.31
	XGBoost	C1	0.6370	4.46	0.94	2.16	0.1998	29.27	2.23	5.41
		C2	0.9623	0.46	0.22	0.68	0.9539	1.68	0.39	1.29
		C3	0.7757	4.75	0.73	2.18	0.3590	23.44	1.96	4.84
		C4	0.9700	0.36	0.21	0.60	0.9549	1.65	0.39	1.28
	SVR	C1	0.3512	7.97	1.08	2.82	0.0047	36.40	2.49	6.03
		C2	0.9201	0.98	0.27	0.99	0.9115	3.23	0.47	1.79
		C3	0.4870	6.30	0.94	2.51	0.2473	27.53	2.16	5.24
		C4	0.9192	0.99	0.27	0.99	0.9090	3.32	0.50	1.82

**Table 5:** Statistics on snow depth prediction performance of deep learning in Mohe.

Station	DP Models	Combinations	Training Set				Testing Set			
			R^2^	MSE	MAE	RMSE	R^2^	MSE	MAE	RMSE
Mohe	1D CNN	C1	0.6770	25.24	3.13	5.02	0.4595	70.56	4.62	8.40
		C2	0.9868	1.03	0.42	1.01	0.9878	1.59	0.57	1.26
		C3	0.6932	23.97	2.97	4.89	0.5185	62.85	4.40	7.92
		C4	0.9849	1.17	0.64	1.08	0.9871	1.68	0.73	1.29
	LSTM	C1	0.6817	24.87	3.27	4.98	0.0544	123.45	6.45	11.11
		C2	0.9802	1.54	0.57	1.24	0.9734	3.47	0.86	1.86
		C3	0.6879	24.39	3.10	4.93	0.0734	120.97	6.26	10.99
		C4	0.9848	1.19	0.48	1.09	0.9848	1.98	0.68	1.41
	1D CNN-LSTM	C1	0.6560	26.88	3.87	5.18	0.1208	114.78	6.79	10.71
		C2	0.9812	1.47	0.78	1.21	0.9803	2.56	0.98	1.60
		C3	0.6672	26.01	3.48	5.10	0.1493	111.06	6.41	10.53
		C4	0.9811	1.47	0.55	1.21	0.9678	4.21	0.93	2.05

**Table 6:** Statistics on snow depth prediction performance of deep learning in Mishan.

Station	DP Models	Combinations	Training Set				Testing Set			
			R^2^	MSE	MAE	RMSE	R^2^	MSE	MAE	RMSE
Mishan	1D CNN	C1	0.3791	7.63	1.25	2.76	0.2128	28.80	2.31	5.37
		C2	0.9369	0.77	0.36	0.88	0.9617	1.40	0.40	1.18
		C3	0.5371	5.69	1.13	2.39	0.5108	17.90	1.80	4.23
		C4	0.9378	0.76	0.35	0.87	0.9471	1.94	0.54	1.39
	LSTM	C1	0.3905	7.49	1.27	2.74	0.2960	25.75	2.16	5.07
		C2	0.9347	0.80	0.33	0.90	0.9592	1.49	0.39	1.22
		C3	0.5241	5.85	1.30	2.42	0.5348	17.02	1.90	4.13
		C4	0.9371	0.77	0.37	0.88	0.9600	1.46	0.43	1.21
	1D CNN-LSTM	C1	0.3816	7.60	1.51	2.76	0.2796	26.36	2.42	5.13
		C2	0.9282	0.88	0.37	0.94	0.9508	1.80	0.51	1.34
		C3	0.5279	5.80	11.27	2.41	0.5320	17.12	1.90	4.14
		C4	0.9230	0.95	0.54	0.97	0.9492	1.86	0.67	1.36

## Data Availability

The data presented in this study are available on request from the corresponding author.
